# pH-Responsive Cross-Linked Low Molecular Weight Polyethylenimine as an Efficient Gene Vector for Delivery of Plasmid DNA Encoding Anti-VEGF-shRNA for Tumor Treatment

**DOI:** 10.3389/fonc.2018.00354

**Published:** 2018-09-25

**Authors:** Xiaoming Li, Xiaoshuang Guo, Yuan Cheng, Xiaotian Zhao, Zhiwei Fang, Yanli Luo, Shujun Xia, Yun Feng, Jianjun Chen, Wei-En Yuan

**Affiliations:** ^1^Engineering Research Center of Cell and Therapeutic Antibody, Ministry of Education, School of Pharmacy, Shanghai Jiao Tong University, Shanghai, China; ^2^Department of Pathology, Shanghai Jiao Tong University Affiliated Sixth People's Hospital, Shanghai, China; ^3^Department of Ultrasound, Rui Jin Hospital Shanghai Jiao Tong University School of Medicine, Shanghai, China; ^4^Department of Respiration, Institute of Respiratory Diseases, School of Medicine, Ruijin Hospital, Shanghai Jiao Tong University, Shanghai, China; ^5^Guangdong Provincial Key Laboratory of New Drug Screening, School of Pharmaceutical Sciences, Southern Medical University, Guangzhou, China

**Keywords:** nanoparticles, polyethylenimine, gene vector, anti-VEGF, tumor treatment

## Abstract

RNA interference (RNAi) is a biological process through which gene expression can be inhibited by RNA molecules with high selectivity and specificity, providing a promising tool for tumor treatment. Two types of molecules are often applied to inactivate target gene expression: synthetic double stranded small interfering RNA (siRNA) and plasmid DNA encoding short hairpin RNA (shRNA). Vectors with high transfection efficiency and low toxicity are essential for the delivery of siRNA and shRNA. In this study, TDAPEI, the synthetic derivative of low-molecular-weight polyethylenimine (PEI), was cross-linked with imine bonds by the conjugation of branched PEI (1.8 kDa) and 2,5-thiophenedicarboxaldehyde (TDA). This biodegradable cationic polymer was utilized as the vector for the delivery of plasmid DNA expressing anti-VEGF-shRNA. Compared to PEI (25 kDa), TDAPEI had a better performance since experimental results suggest its higher transfection efficiency as well as lower toxicity both in cell and animal studies. TDAPEI did not stimulate innate immune response, which is a significant factor that should be considered in vector design for gene delivery. All the results suggested that TDAPEI delivering anti-VEGF-shRNA may provide a promising method for tumor treatment.

## Introduction

Angiogenesis is an essential prerequisite for the growth and spread of tumors since abundant blood supply is needed to get adequate oxygen and other essential nutrients for tumors to grow rapidly (1, 2). Tumors often induce angiogenesis by secreting various growth factors such as vascular endothelial growth factor (VEGF) (3, 4). In many studies, over expression of VEGF has proven to be a critical factor contributing to tumor angiogenesis (5, 6). Hence, the inhibition of VEGF expression has been a therapeutic strategy for tumor treatment.

RNA interference (RNAi) is a biological process through which gene expression can be inhibited by RNA molecules with high selectivity and specificity, providing a promising tool for tumor treatment (7–9). RNAi techniques overcome the disadvantages of chemotherapy (10), a traditional way for tumor treatment, in which normal cells and tumor cells cannot be distinguished effectively and chemotherapy is often with severe side effects (11, 12). Many studies have confirmed that RNAi can specifically silence cancer-related genes to inhibit tumor growth (13–15), angiogenesis (16–18), chemoresistance (19, 20) and metastasis (13, 21, 22).

Two types of molecules are often applied to inactivate the expression of target genes: synthetic double stranded small interfering RNA (siRNA) and plasmid DNA encoding short hairpin RNA (shRNA). For siRNA mediated RNAi, siRNA is delivered into the cytoplasm and then RNA-induced silencing complex (RISC) formed (23). After that, RISC binds to target mRNA by intermolecular expression of base pairing, contributing to cleavage and degradation of target mRNA (24). As for plasmid DNA encoding shRNA, it is transported into the cell nucleus for transcription of pre-shRNA. The formed pre-shRNA will be transported to the cytoplasm by exportin 5, processed by Dicer to form mature shRNA and loaded into RISC to play the role of gene silencing (25).

Efficacy of RNAi tumor treatments is partially dependent on the choice of gene vectors. Viral vectors are commonly employed for laboratory delivery of shRNA due to their high transfection efficiency. However, problems of immunogenicity and toxicity have limited their applications (26–28). In contrast, cationic polymers, a kind of non-viral vector, have no immunogenicity and are easier to be chemically modified for targeted gene delivery. However, the low transfection efficiency remains a problem to be solved for this system. In recent years, polycation vectors have been well studied by many researchers (29–31). Polyethyleneimine (PEI) is a kind of cationic polymer regarded as an effective transfection agent with high buffering effect due to the existence of protonated amine groups in the structure, which can enhance the “proton sponge effect” and accomplish the tasks of gene condensation, cellular uptake, endosome escape and release of therapeutic genes. The transfection efficiency and toxicity of PEI are positively correlated with molecular weight, so high transfection efficiency is accompanied by high toxicity (32, 33). To get a safe and efficient cationic polymer, a biodegradable PEI derivative named TDAPEI was synthesized by conjugating branched PEI 1.8 kDa with 2,5-thiophenedicarboxaldehyde (TDA). The formed imine bond is liable to be degraded in low pH environment theoretically, which could significantly lower the biological toxicity of polymers.

In our study, the cationic polymer TDAPEI was synthesized and characterized by fourier transform infrared spectroscopy (FTIR) and proton nuclear magnetic resonance (^1^H NMR). We then further investigated the characteristics of TDAPEI/pDNA complexes, including condensation ability, particle size, zeta potential and morphology. Transfection efficiency, intracellular uptake, cytotoxicity and innate immune response were tested *in vitro*. We evaluated the therapeutic effect and *in vivo* toxicity in tumor bearing mice.

## Materials and methods

### Materials

Branched PEI (1.8 and 25 kDa), N,N-Dimethylformamide (DMF), agarose and dimethyl sulfoxide were acquired from Sigma-Aldrich (St Louis, MO, USA). 2,5-thiophenedicarboxaldehyde (TDA) was purchased from Meryer (Shanghai) Chemical Technology Co., Ltd. Dialysis bags made by cellulose membrane (MWCO = 10 kDa) were acquired from Thermo Fisher Scientific. A milli-Q instrument (Millipore) was used to purify water. Plasmid DNA expressing mouse-VEGF-shRNA and GFP was constructed by PHY-310 vector (Bioroot Biology, Shanghai, China). The single strain oligonucleotides sequence was 5′- GATCCGATGTGAATGCAGACCAAAGAATTCAAGAGATTCTTTGGTCTGCATTCACATTTTTTTG-3′. Plasmid extraction kits were purchased from Bioteke Corporation. CT26.WT and SMMC7721 cells were purchased from the Cell Bank of Chinese Academy of Sciences (Shanghai, China). The complete medium for cells was composed of 90% Roswell Park Memorial Institute (RPMI) 1640 Medium (Media Tech, Herndon, VA, USA), 9% fetal bovine serum (FBS; HyClone, Logan, UT, USA) and 1% penicillin-streptomycin solution (Gibco, Grand Island, N.Y.). Cell Counting Kit-8 (CCK-8) was purchased from Dojindo Molecular Technologies, Inc. BALB/c mice were obtained from Shanghai Slac Laboratory Animal Co., Ltd.

### Synthesis and characterization of TDAPEI

The synthesis of TDAPEI was performed according to the process previously reported in our published paper (34–40). In brief, 1 mmol PEI (1.8 kDa) was added to 30 mL anhydrous DMF and stirred vigorously to be dissolved. 2 mmol TDA was dissolved in 30 mL anhydrous DMF and then dropwisely added to the PEI solution with vigorous stirring at room temperature for 24 h. The solvent was removed by reduced pressure rotary evaporation. The sticky residue was re-dissolved in deionized water. Dialysis bags made by cellulose membrane (MWCO = 10 kDa) were used to remove small fragments for another 48 h. The yellowish spongy product, TDAPEI, was obtained after 48 h of lyophilization. The final product was stored at −20°C for later use. The structure of TDAPEI was confirmed by fourier transform infrared spectroscopy (FTIR) and proton nuclear magnetic resonance (^1^H NMR). The molecular weight of TDAPEI was determined by high performance size exclusion chromatography (SEC). A series of polyethylene glycol (PEG) standards were used for the standard curve and PEI 25k Da for calibration. The M_w_ and polydispersity index (PDI) were calculated by the software Agilent GPC-Addon. To assess the degradability, TDAPEI was dissolved in FORMIC buffer solutions of different pH values (pH = 7.4, 6.0, 5.0) respectively and incubated at 37°C for 72 h. The samples were collected at certain time points and the average molecule weight was determined by SEC. The three pH values were selected to simulate the environment of blood plasma, endosomes and lysosomes, respectively.

### Preparation of TDAPEI/pDNA polyplexes

TDAPEI was dissolved in ultrapure water and diluted into 2 mg/mL. pDNA was diluted into 20 μg/mL as stock solutions. The TDAPEI/pDNA polyplexes were prepared in various weight/weight (w/w) ratios. The TDAPEI stock solution (2 mg/mL) was diluted into certain concentrations and added to pDNA stock solution (20 μg/mL). The mixture was thoroughly mixed by pipetting for 30 times and then incubated at room temperature for 30 min to obtain self-assembled polyplexes. PEI (25 kDa)/pDNA polyplexes at 2 w/w ratio were prepared with the same method, which is a classical positive control group in many gene delivery studies (34, 38, 41).

### Agarose gel electrophoresis

Agarose gel electrophoresis was used to evaluate the stability of the TDAPEI/pDNA polyplexes. Polyplexes at various w/w ratios were respectively mixed with 6 × loading buffer containing GelRed (30 mM EDTA, 36% (v/v) Glycerol, 0.05% (w/v) Bromophenol Blue and 0.05%(w/v) Xylene Cyanol FF). Naked pDNA solution stained with GelRed was set as a negative control. Then these different mixtures were loaded into 1% (w/v) agarose gel. Electrophoresis was carried out with 1 × TAE running buffer at 110 V for 45 min. The stripes of DNA were visualized by UV transilluminator (Gel Imaging System, Tanon-3500).

### Physicochemical characterization of TDAPEI/pDNA polyplexes

The particle size and polydispersity index (PDI) of the TDAPEI/pDNA polyplexes at various w/w ratios were determined by dynamic light scattering (90 Plus Particle Size Analyzer, Brookhaven Instruments Corporation, NY, USA) at room temperature. Zeta potential of polyplexes was measured at room temperature by Zetasizer Nano ZSP (Malvern Instruments, UK). All measurements were repeated for three times. Morphology of TDAPEI/pDNA polyplexes (w/w ratio = 10) was observed with a transmission electron microscope (JEOL JEM-2010 TEM) at an acceleration voltage of 200 kV.

### *In vitro* cell transfection

Human hepatocarcinoma cells SMMC7721 and mouse colon adenocarcinoma cells CT 26.WT acquired from Cell Bank of the Chinese Academy of Sciences were used to determine *in vitro* cell transfection efficiency of TDAPEI/pDNA polyplexes. Cells were cultured in RPMI-1640 complete medium at 37°C in a 5% CO_2_ moist atmosphere. Cells were digested by trypsin and diluted to 5–10 × 10^4^/mL with RPMI-1640 complete medium. Then the cells were seeded in 48-well plates, with 500 μL dilute solution per well, and cultured for 24 h at 37°C in a 5% CO_2_ moist atmosphere. The culture medium was removed and the cells were washed with PBS for three times. 250 μL RPMI-1640 medium and 50 μL polyplexes of various w/w ratios (the pDNA mass is 0.5 μg per well) were added into each well and the cells were incubated for 4 h. Meanwhile, naked pDNA was used as negative control and PEI (25 kDa)/pDNA polyplexes at 2 w/w ratio (optimal mass ratio) was prepared as positive control group, which is currently the gold standard for *in vitro* transfection. The culture medium was replaced by RPMI-1640 complete medium and incubated for another 44 h. Transfection efficiency is directly observed by fluorescence microscope (Olympus, Tokyo, Japan) and quantitatively measured by flow cytometry (BD FACSCalibur).

### *In vitro* expression of VEGF-A

The *in vitro* expression of VEGF-A was tested in CT26 cells. The cells were seeded in 48-well plates and incubated for 24 h at 37°C in a 5% CO2 moist atmosphere. Then, the cells were respectively treated with TDAPEI/pDNA polyplexes (w/w = 20), PEI (25 kDa)/pDNA polyplexes (w/w = 2), naked pDNA and PBS for 4 h. The culture medium was replaced by RPMI-1640 complete medium and incubated for another 44 h. The culture supernatant of cells was centrifuged and analyzed by Mouse VEGF-A ELISA kit (DKW12-2734-048) to determine the *in vitro* expression of VEGF-A.

### *In vitro* cytotoxicity

The *in vitro* cytotoxicity assay was conducted in SMMC7721 and CT26 cells by CCK-8 method. The two kinds of cells (1 × 10^4^/well) were respectively seeded in 96-well plates and incubated for 24 h at 37°C in a 5% CO_2_ moist atmosphere. The culture medium was removed and cells were washed by PBS for three times. 50 μL RPMI-1640 medium and 10 μL PBS (negative control), PEI (25 kDa)/pDNA polyplexes (positive control), and TDAPEI/pDNA complexes at various w/w ratios were respectively added into each well and the cells were incubated for 4 h. Then, 10 μL CCK-8 was added into each well. The plate was incubated in 37°C, 5% CO_2_ incubator for 2 h. Auto Microplate Reader (Spectra Max M3 Multi- Mode) was used to read the absorption value under the wavelength of 450 and 630 nm. The percentage of cell viability was calculated as follow.

$$\begin{array}{l} \begin{array}{l} \text{Cell viability}(\%)=\frac{\text{Sample}(\text{OD}450)-\text{Sample}(\text{OD}630)}{\text{Normal cell}(\text{OD}450)-\text{Normal cell}(\text{OD}630)}\times 100\% \end{array} \end{array}$$

### Innate immune response test

The innate immune response caused by TDAPEI/pDNA polyplexes was tested in murine macrophage RAW264.7 cells. The cells were cultured in Dulbecco's Modified Eagle Medium (DMEM) with 10% FBS and seeded on 6-well plates. When the cell density reached about 85%, the culture medium was replaced by fresh DMEM and cells were respectively treated with TDAPEI/pDNA polyplexes (w/w = 20), PEI (25 kDa)/pDNA polyplexes (w/w = 2) and naked pDNA for 4 h. Then, the polyplexes and pDNA were removed and the cells were incubated in DMEM with 10% FBS for another 24 h. The culture supernatant of cells was centrifuged and analyzed by Mouse IL-6 ELISA kit (DKW12-2060-096) and Mouse TNF-α ELISA kit (DKW12-2720-096).

### *In vivo* treatment in tumor model mice

To assess the tumor treatment efficacy of TDAPEI/pDNA polyplexes, CT26 tumor model mice were established. The 5-week old female BALB/c mice, weighed 20 ± 2 g, were housed in Specific Pathogen Free (SPF) environment for 1 week before the animal study.

To establish *in vivo* tumor models, 0.1 ml CT26 cell suspension (5 × 10^6^/ml) was subcutaneously injected to the mice at the right armpit. The mice were kept in SPF environment until the tumor volume reached about 200 mm^3^. Then, the mice were divided into 4 groups randomly (6 mice in each group): blank group (saline), negative control group (naked pDNA), positive control group (PEI (25 kDa)/pDNA polyplexes) and experimental group (TDAPEI/pDNA polyplexes). The mice were treated by intra-tumor injection of 0.1 mL saline or therapeutic solutions containing 10 μg pDNA. The injection was given every 3 days. The shortest diameters (width, W) and longest diameters (length, L) of tumors were measured by vernier caliper to calculate the tumor volume. The formula is: V = W^2^ × L/2. At the 14th day after the first injection, the mice were sacrificed and the tumors were separated and analyzed.

### Microvessel quantification

To assess the anti-angiogenesis effect of the polyplexes, microvessel quantity was measured by CD31 immuno-histochemical staining. At the 14th day after the first injection, the separated tumors were fixed in 4% paraformaldehyde for 24 h and made into paraffin-embedded samples and then sectioned. The sections were stained with anti-CD31 antibody to mark microvessels. The sections were photographed by optical microscope and the images were analyzed by Image-Pro Plus software to get the number of positively stained microvessels.

### *In vivo* expression of VEGF-A

To identify the silence efficiency of polyplex in tumor tissue, we measured the concentration of expressed VEGF-A protein in tumor tissue homogenate at the 14th day after the first injection by using Mouse VEGF-A ELISA Kit (DKW12-2734-048).

### *In vivo* toxicity

Histological examination was adopted to demonstrate the toxicity of polyplexes to main organs. The organs were removed at the 14th day after the first injection and fixed in 4% paraformaldehyde. Hematoxylin and eosin (H&E) was used to stain organ sections and the slides were observed by optical microscope to observe the lesion.

### Ethics statement

All the experiments followed the Regulations for the Administration of Affairs Concerning Experimental Animals (China, 2014) and the National Institutes of Health Guide for Care and Use of Laboratory Animals (GB14925-2010). The Committee for Laboratory Animal Ethics of Shanghai Jiao Tong University approved the experiments.

### Statistical analysis

The data were presented as mean ± standard deviation of replicates. Analysis of variance (ANOVA) and independent samples *t*-test was performed and a value for ^*^P < 0.05, ^**^P < 0.01, and ^***^P < 0.001 was considered statistically significant.

## Results and discussion

### Synthesis and characterization of TDAPEI

**Figure 1:**
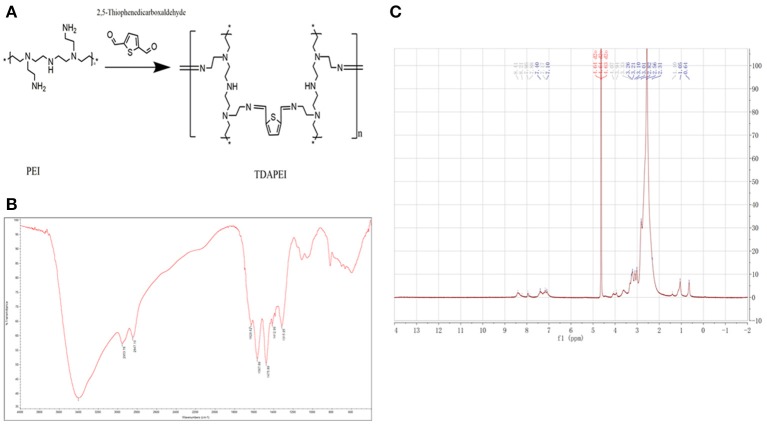
Synthesis and characterization of TDAPEI. **(A)** Chemical structure and polymerization reaction of TDAPEI. **(B)** FTIR spectrum of TDAPEI. **(C)** ^1^HNMR spectrum of TDAPEI.

Results of FTIR and ^1^H NMR of TDAPEI were shown in Figure 1. TDAPEI is formed by polymerization of branched PEI (1.8 kDa) and TDA through imine bonds (Figure 1A). Characteristic IR adsorption peak of imine bonds is at 1690-1590 cm^−1^ and a moderate adsorption peak at 1629.62 cm^−1^ was shown in the spectrum, which indicated that imine bonds were formed and the original aldehyde group (characteristic IR adsorption peak 1755-1665 cm^−1^) disappeared (Figure 1B). The ^1^H NMR spectrum of TDAPEI in D_2_O was consistent with our expectation. Signal at δ = 7.96-8.41 ppm indicated the presence of methyne protons close to imino groups, while signal at δ = 10 ppm disappeared, which indicated that aldehyde group in TDA completely reacted with amino and formed imine bonds. The FTIR spectrum and ^1^H NMR spectrum of TDAPEI were in consistent and confirmed the successful synthesis of TDAPEI.

**Figure 2:**
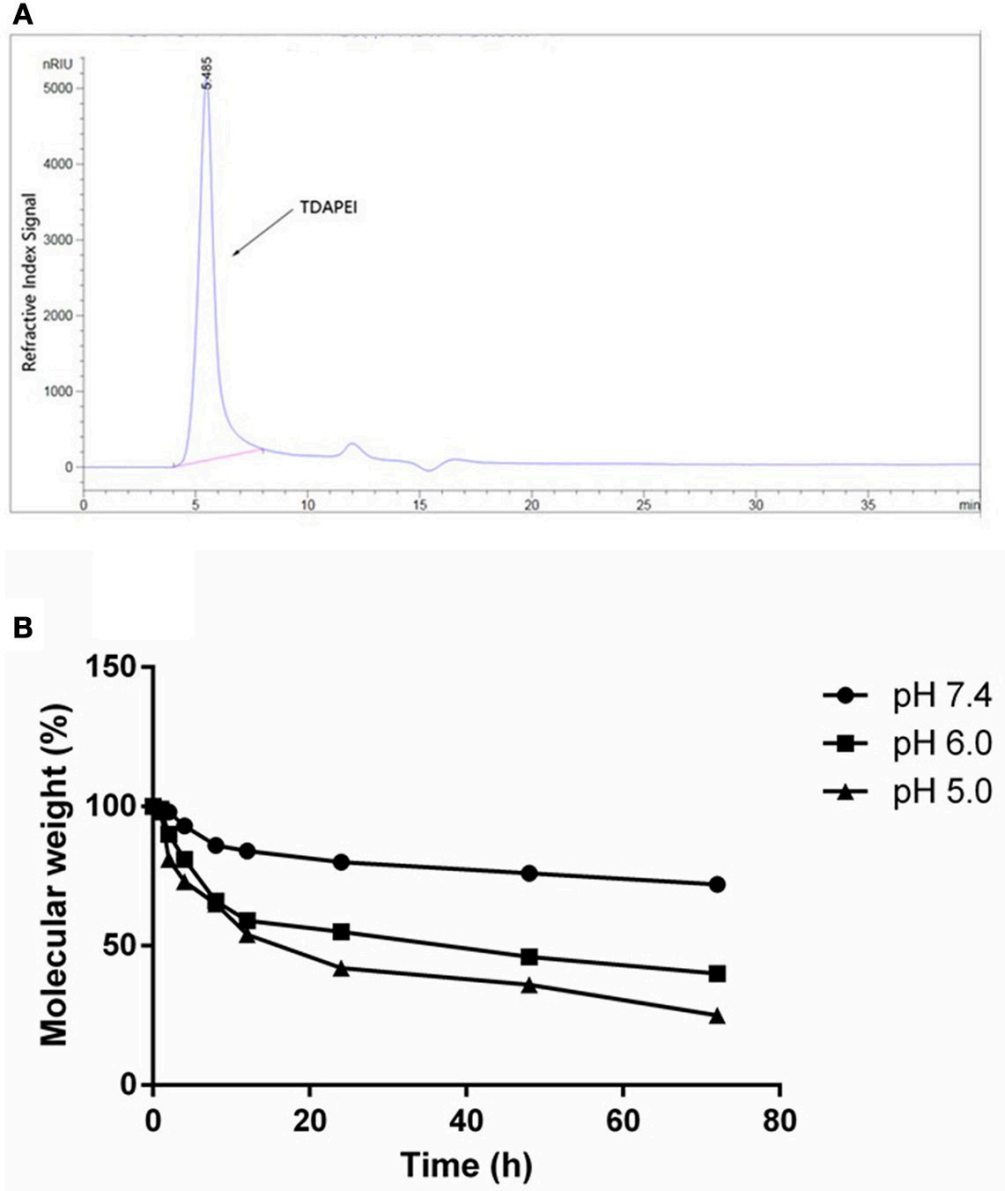
**(A)** GPC spectrum of TDAPEI. **(B)** pH-responsive degradation of TDAPEI. TDAPEI was dissolved in FORMIC buffer solutions of different pH values (pH = 7.4, 6.0, 5.0) respectively and incubated at 37°C, followed by SEC tests. The molecular weights of the degraded polymers were determined as the weights measured by SEC test relative to the original molecular weight of TDAPEI (100%).

The GPC spectrum of TDAPEI was shown in Figure 2A. M_w_ of polymer TDAPEI was calculated to be 23.2k Da, and the PDI was 1.65. As shown in Figure 2B, TDAPEI degraded at different rates in the three solutions with different pH values (pH = 7.4, 6.0, 5.0). The degradation rate was relatively high in an acidic environment and the polymer remained stable in a neutral environment.

### Agarose gel electrophoresis

**Figure 3:**
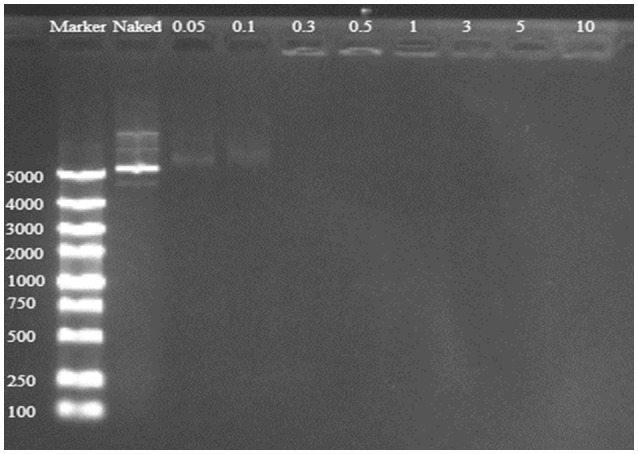
TDAPEI/pDNA agarose gel electrophoresis. 100-5000bp marker was loaded in the first lane and naked DNA was in the second lane. The polyplexes were at 0.05, 0.1, 0.3, 0.5, 1, 3, 5, and 10 w/w ratios.

Good binding and condensation ability is a prerequisite for a gene carrier to protect gene from degradation. To determine the ability of binding and condensing pDNA of TDAPEI and the stability of TDAPEI/pDNA polyplexes, agarose gel electrophoresis test was performed. TDAPEI/pDNA polyplexes at w/w ratios of 0.05, 0.1, 0.3, 0.5, 1, 3, 5, and 10 were chosen. As shown in Figure 3, with the increase of w/w ratio, the stripes of pDNA started to disappear at 0.3 w/w ratio, which indicated that the migration of pDNA in electric field was retarded in this case and pDNA was completely bound and wrapped by TDAPEI. A conclusion could be made that the polymer TDAPEI had a strong ability to condense pDNA into tight polyplexes to protect pDNA from nuclease.

### Physicochemical characterization of TDAPEI/pDNA polyplexes

**Figure 4:**
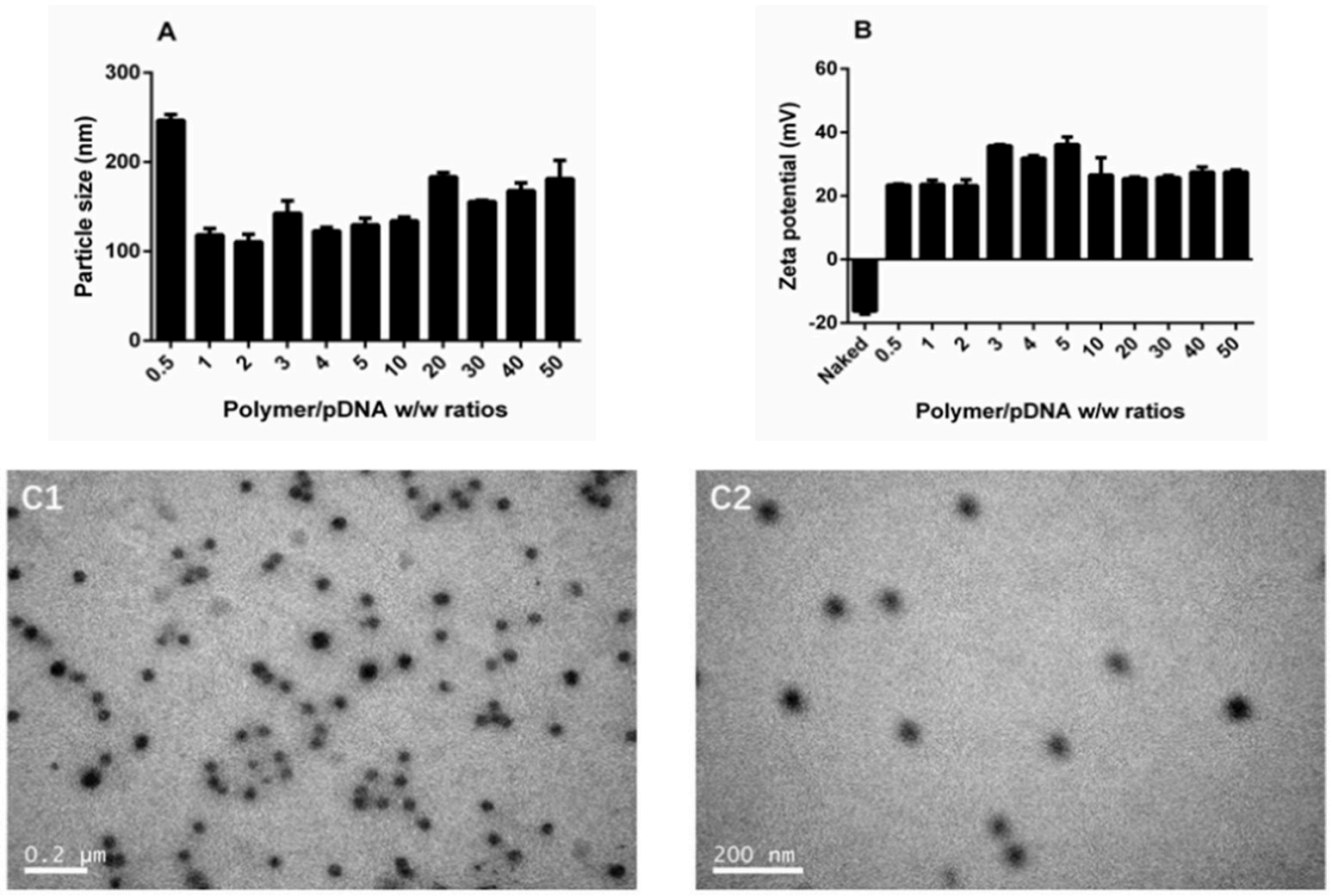
Characterization of TDAPEI/pDNA complexes. Particle size **(A)** and Zeta potential **(B)** of TDAPEI/pDNA polyplexes at 0.5,1,2,3,4,5,10, 20, 30, 40, and 50 w/w ratios in water. **(C)** TEM images of polyplexes at w/w ratio of 10.

The DNA binding and condensing ability are closely related to the particle size and zeta potential of polyplexes, which also has significant impacts on cell endocytosis and gene transfection. The ideal particle size for cell endocytosis is about 20–200 nm. In our study, particle size of TDAPEI/pDNA complexes at various w/w ratios ranging from 0.5 to 50 were measured by 90 Plus Particle Size Analyzer. As is shown in Figure 4A, the particle size was 246.5 ± 6.7 nm at 0.5 w/w ratio. When the mass ratio increased to 10, the particle size of polyplexes decreased and reached a stable value around 130 nm, implying the tight pDNA condensation at this ratio. Theoretically, increased number of protons captured by the polymer enhanced the DNA binding and condensing ability of polymers. The DNA condensing ability was positively related to the surface charge density, so the particle size increased. On the other hand, higher concentration of TDAPEI caused a larger occupation in volume, which also contributed to the larger particle size. Particle sizes ranged from 110.5 to 183.4 nm and PDI were all lower than 0.3 (w/w ratio 1~50), which indicated the polyplexes had a narrow dispersity and kept stable in this environment.

The zeta potential of polyplexes has an important effect on gene transfection efficiency and cytotoxicity. The surface of cell membrane is negatively charged, so it is difficult for naked DNA to cross cell membrane barrier. Particles with positive charge can cross cell membrane barrier but high cationic charge density may lead to the disruption of the cell membrane, which is responsible for the high cytotoxicity. As shown in Figure 4B, the zeta potential of polyplexes at different w/w ratios ranged from 23.2 to 36.1 mV and stabilized around 27 mV. The changing trend of zeta potential was in accordance with particle size and can be explained with similar reason. At low w/w ratios, more polymer brought higher positive charge density, resulting in the increase of zeta potential. Then the pH value rose, which in turn contributed to the decrease of positive charge. The result indicated that the disperse system was stable and implied the possibility for gene transfection.

The morphology of polyplexes at 10 w/w ratio observed by transmission electron microscopy is shown in Figure 4C. The polyplexes had uniform size and a spherical shape, which was in accordance with the results tested by Particle Size Analyzer.

### *In vitro* cell transfection

**Figure 5:**
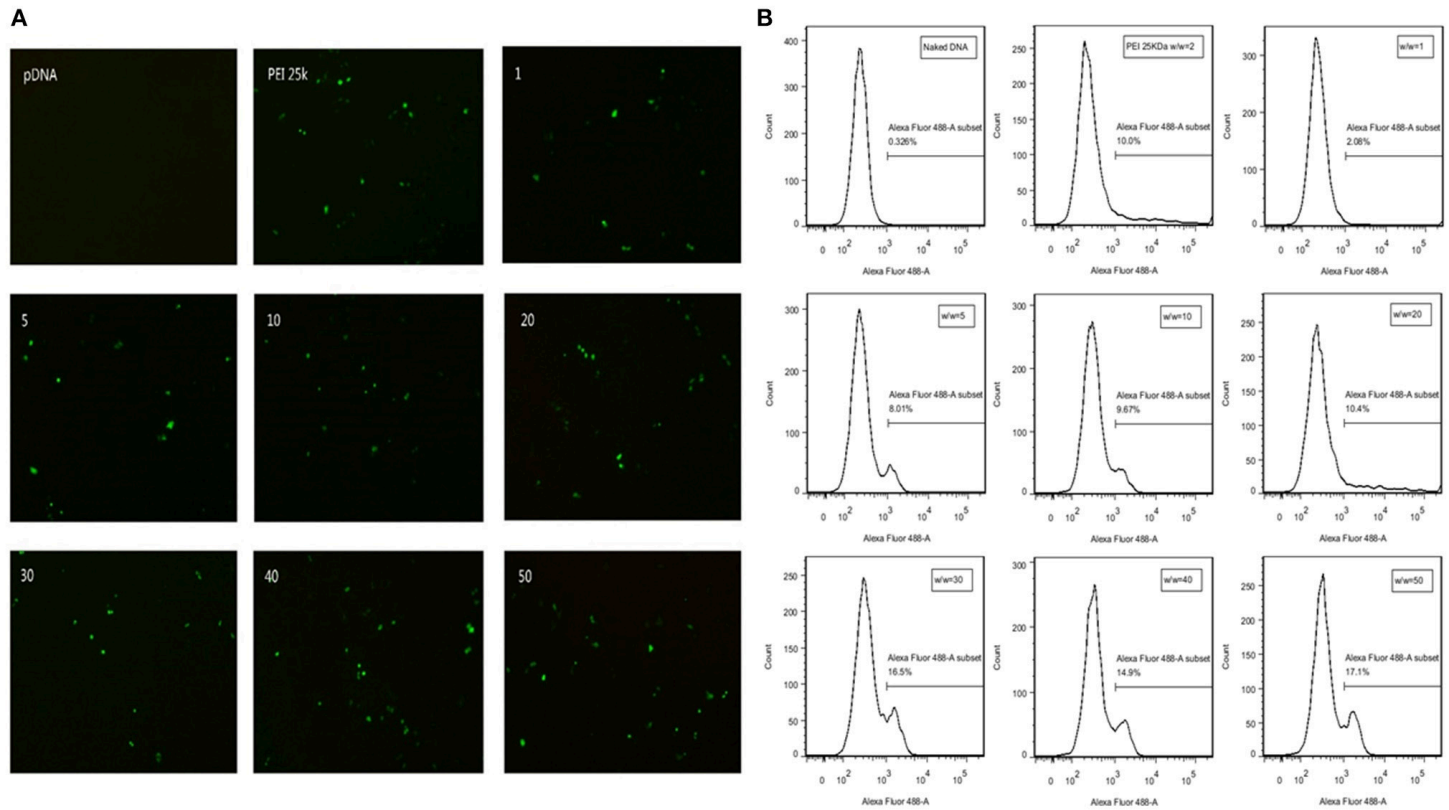
**(A)** GFP expression in SMMC7721 cells transfected with naked pDNA, PEI 25k Da/pDNA (w/w ratio = 2), TDAPEI/pDNA complexes at w/w ratios of 1,5,10, 20, 30, 40, and 50. **(B)** Transfection efficiency in CT26 cells quantified by GFP-expressing cells using the flow cytometer.

GFP expression vector was constructed in our plasmid DNA. Cells successfully transfected by polyplexes could express GFP protein and thus emit fluorescence at wavelength of 488 nm. Transfection efficiency is directly observed by fluorescence microscope in SMMC7721 cells (Figure 5A) and quantitatively measured by flow cytometry in CT26 cells (Figure 5B). There is almost no fluorescence in the naked DNA group, which means naked DNA could not get into cells due to its instability and negative charge. Hence, positive charge of the polymer played an important role in entering the cells. At high w/w ratios, the transfection efficiency of TDAPEI/pDNA poplyplexes was comparable to or even higher than PEI (25 kDa)/pDNA polyplexes and no significant change in morphology of cells was observed in TDAPEI/pDNA poplyplexes treated groups. We believe the high transfection efficiency of these groups was owing to the better ability in releasing pDNA during the endosomal escape, as has been discussed in the previous study. Ur, et al. (42) High-molecular-weight PEI has a high density of positive charge, which is advantageous in crossing cell membrane barrier. However, after rupturing the endosomes through “proton sponge effect,” it is more difficult for high-molecular-weight PEI to unbind pDNA than that with low molecular weight, such as PEI (1.8 kDa) used in our polymer design. Before entering the cells, TDAPEI was of high molecular weight, thus it provided comparable positive charge density to that of PEI (25 kDa). The advantage of our constructed polycation TDAPEI is that it could be degraded in low acidic medium. The imine linkages broken down and the polymer was metabolized into PEI (1.8 kDa) fragments. After that, the binding ability greatly weakened and it was easier for the polyplex to release pDNA, which led to higher transfection efficiency.

### *In vitro* expression of VEGF-A

**Figure 6:**
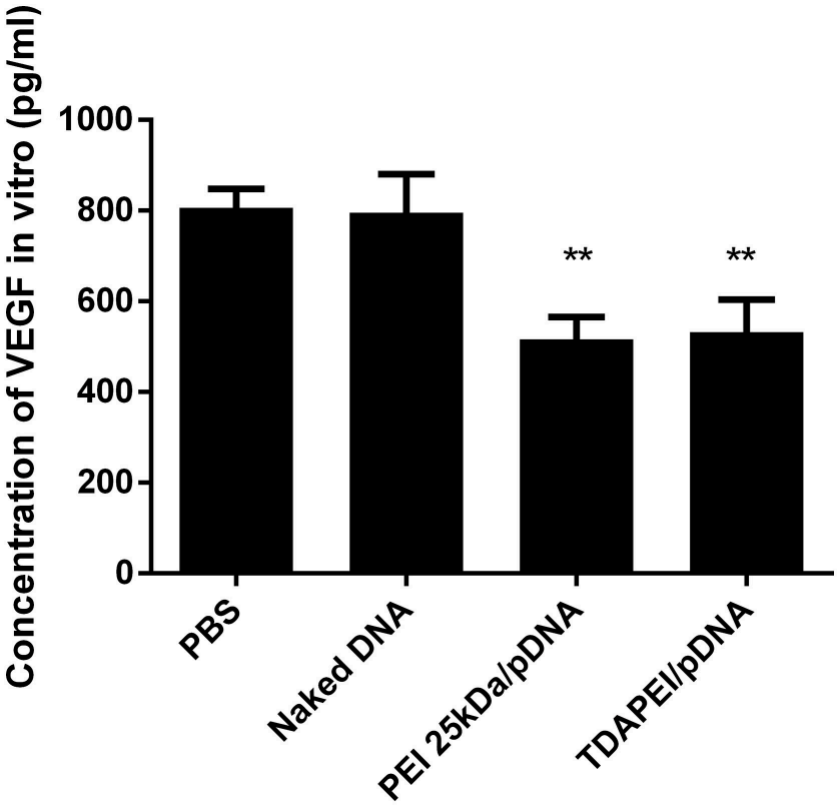
*In vitro* expression of VEGF-A in CT26 cells treated with TDAPEI/pDNA polyplexes (w/w = 20), PEI (25 kDa)/pDNA polyplexes (w/w = 2), naked pDNA and PBS. Data were displayed as mean ± SD. (*n* = 6). **P < 0.01.

The expression level of VEGF-A in CT26 cells was determined by ELISA. As shown in Figure 6, compared with PBS group and naked DNA group, the expression level of VEGF-A was significantly lower in TDAPEI/pDNA poplyplexes and PEI (25 kDa)/pDNA poplyplexes treated groups (^***^p < 0.001, ^**^p < 0.01). TDAPEI/pDNA poplyplexes had equally excellent silence efficiency compared with PEI (25 kDa)/pDNA poplyplexes.

### *In vitro* cytotoxicity

**Figure 7:**
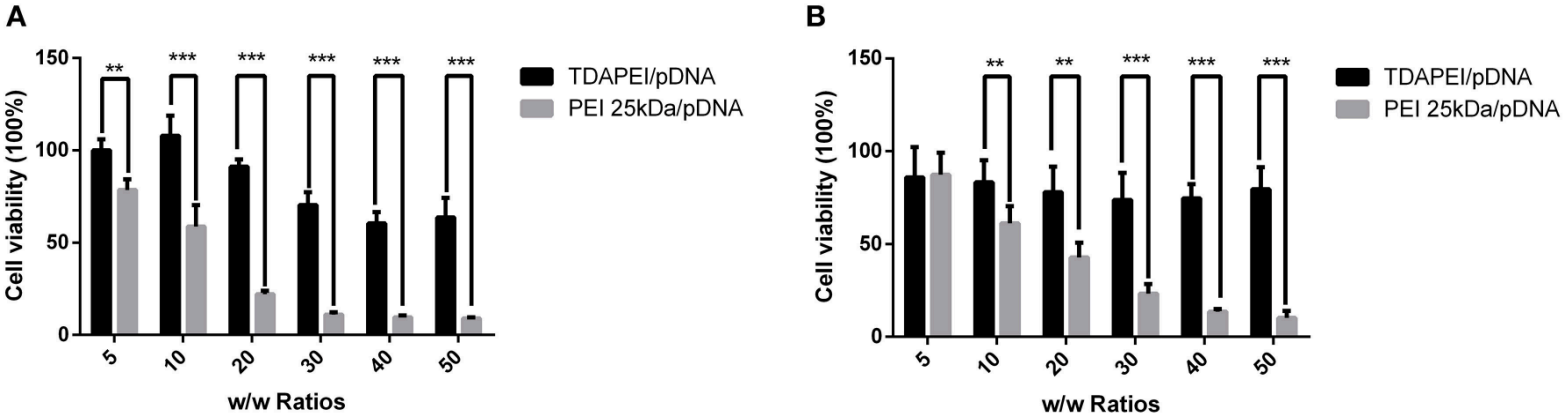
Viability of SMMC **(A)** and CT26 cells **(B)** transfected by TDAPEI/pDNA complexes and PEI 25kDa/pDNA complexes at w/w ratios of 5, 10, 20, 30, 40, and 50. Data were displayed as mean ± SD. (*n* = 6). **P < 0.01, ***P < 0.001.

*In vitro* cytotoxicity of TDAPEI/pDNA complexes in SMMC7721 and CT26 cells were evaluated by CCK-8 assay. As is shown in Figure 7, the cell viability of TDAPEI/pDNA complexes in SMMC7721 cells was in the range from 107.98 to 60.47% at w/w ratios of 5, 10, 20, 30, 40, and 50, while that of PEI (25 kDa)/pDNA poplyplexes was from 78.62 to 8.98%. In CT26 cells, the cell viability of TDAPEI/pDNA and PEI (25 kDa)/pDNA polyplexes was from 85.93 to 73.85% and from 87.33 to 10.24%, respectively. In both cells, the cell viability of TDAPEI /pDNA is relatively stable while an apparent decline occurred in PEI (25 kDa)/pDNA poplyplexes group. There was a significant difference in cytotoxicity between TDAPEI/pDNA poplyplexes and PEI (25 kDa)/pDNA poplyplexes groups, especially at high w/w ratios (^***^p < 0.001). The lower cytotoxicity of TDAPEI /pDNA could be explained by its biodegradable quality. Based on the results of *in vitro* cell transfection and cytotoxicity, we chose TDAPEI/pDNA poplyplexes at w/w ratio of 20 in the following animal study.

### Innate immune response test

**Figure 8:**
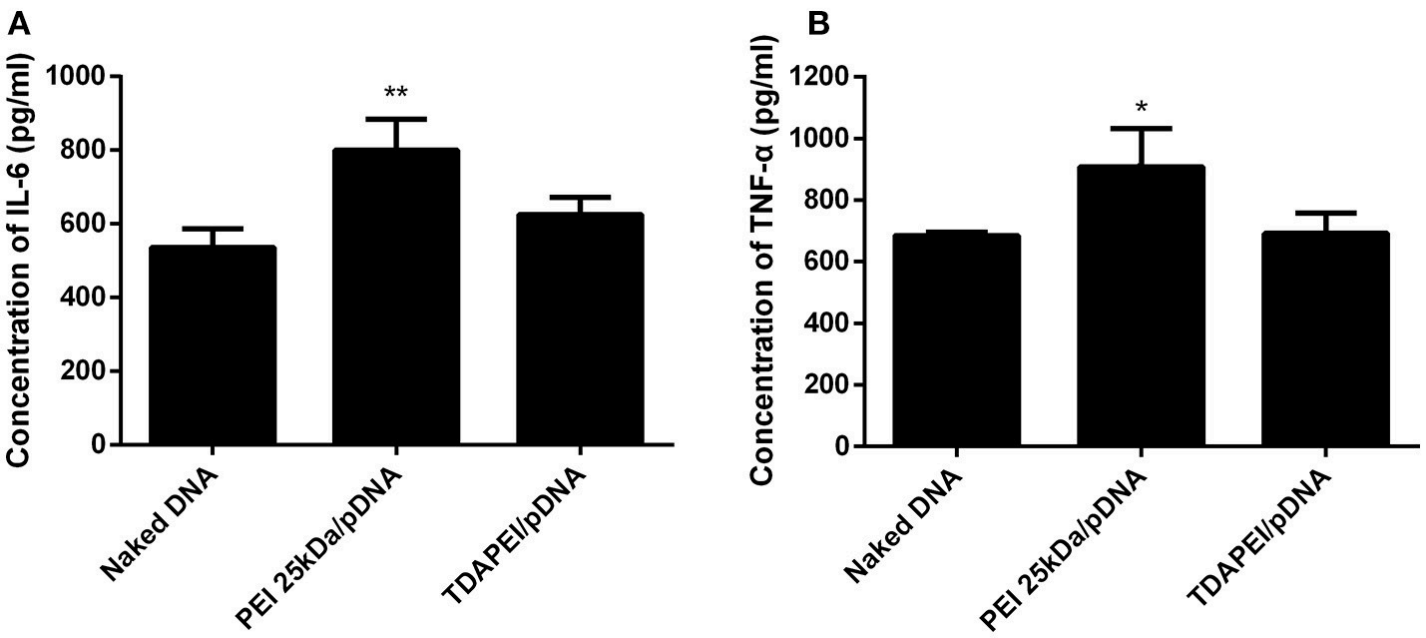
The concentration of IL-6 **(A)** and TNF-α **(B)** secreted in the culture supernatant of murine macrophage RAW264.7 cells. Data were displayed as mean ± SD. (*n* = 6). *P < 0.05, **P < 0.01.

Innate immune response can stimulate the secretion of IL-6 and TNF-α. The level of innate immune response activated by our treatment was evaluated by the concentration of IL-6 and TNF-α in murine macrophage RAW264.7 cells. As is shown in Figure 8, for TDAPEI/pDNA poplyplexes group, the concentrations of IL-6 and TNF-α secreted in the culture supernatant of cells were similar to that in naked DNA group, while the concentrations in PEI 25 kDa group were significantly higher than that in naked DNA group (IL-6: ^**^P < 0.01; TNF-α: ^*^P < 0.05). The result indicated that TDAPEI/pDNA polyplexes (w/w = 20) had a low innate immune response. However, considering the significant heterogeneity of RAW264.7 cells used in this study, further *in vivo* experiments are needed to confirm this preliminary conclusion. We will testify this in future *in vivo* studies.

### *In vivo* anti-tumor treatment

**Figure 9:**
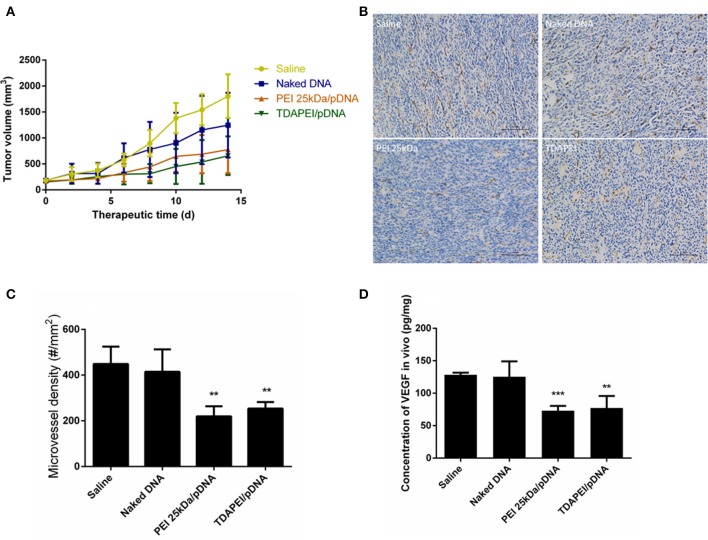
**(A)** Tumor growth curves in CT26 tumor bearing mice treated by saline, naked DNA, PEI 25kDa and TDAPEI/pDNA. Data are displayed as mean ± SD, *n* = 6. **P < 0.01. **(B)** Tumor section images of immunohistochemical staining for CD31 in saline, naked DNA, PEI 25kDa and TDAPEI groups. **(C)** Microvessel density in CT26 tumor bearing mice treated by saline, naked DNA, PEI 25kDa and TDAPEI/pDNA at Day 14. Data are displayed as mean ± SD, *n* = 6. **P < 0.01. **(D)** *in vivo* concentration of expressed VEGF-A protein in tumor tissue homogenate at Day 14. Data are displayed as mean ± SD, n = 6. **P < 0.01.

Plasmid DNA expressing mouse-VEGF-shRNA was used as the antiangiogenic agent in our anti-tumor study. TDAPEI/pDNA poplyplexes at 20 w/w ratio, PEI (25 kDa)/pDNA poplyplexes, naked pDNA and saline were intratumorally injected in four groups of tumor-bearing BALB/c mice. Figure 9A showed the tumor growth curves during the 14-day treatment period. In saline and naked DNA groups, the tumor grew rapidly and no statistical significance in final tumor volume was observed between these two groups (*p* > 0.05), which indicated that naked pDNA had hardly any therapeutic effect on tumor. In TDAPEI/pDNA poplyplexes and PEI (25 kDa)/pDNA poplyplexes treated groups, the growth rates of tumors were similarly low. There existed significant differences for these two groups in final tumor volume compared to that in saline group (TDAPEI: ^***^p < 0.001; PEI 25 kDa: ^**^p < 0.01). Besides, when evaluating the anti-tumor effect of polyplexes *in vivo*, the cytotoxicity of gene vector of polyplexes should be considered as well, which may be a potentially additional contributor to the anti-VEGF therapy. In this case, the more toxic the gene vectors are, the more anti-tumor effects of polyplexes are observed. It is possible that the cytotoxicity of PEI (25 kDa) in this study may finally contribute to the tumor treatment. The final tumor volumes of the four groups were listed as follows: 1,805 ± 171.7 mm^3^ in saline group; 1,247 ± 254.3 mm^3^ in naked pDNA group; 775.7 ± 185.6 mm^3^ in TDAPEI/pDNA poplyplexes group and 657.6 ± 151.9 mm^3^ in PEI (25 kDa)/pDNA poplyplexes group.

To quantitatively evaluate the antiangiogenic effect, tumors were taken out at the last day of treatment period and CD31 immuno-histochemical staining was conducted to determine the number of endothelial cells. The figures of slices were analyzed by Image-Pro Plus. As shown in Figures 9B,C, the microvessel densities in groups of saline, naked pDNA, TDAPEI/pDNA poplyplexes and PEI (25 kDa)/pDNA poplyplexes were: 414.5 ± 40.20/mm^3^, 448.5 ± 31.08/mm^3^, 253.8 ± 11.63/mm^3^, 219.5 ± 18.11/mm^3^. The quantity of microvessels in PEI (25 kDa) and TDAPEI group was significantly lower than that in saline group (^**^p < 0.01). There was no significant difference between PEI (25 kDa) and TDAPEI groups.

To assess the *in vivo* silence efficiency of the poplyplexes, the concentration of expressed VEGF-A protein in tumor tissue homogenate at the 14th day after the first injection was measured by ELISA. As shown in Figure 9D, the expression level of VEGF-A was significantly lower in TDAPEI and PEI (25 kDa groups (^**^p < 0.01) and there was no significant difference between PEI (25 kDa) and TDAPEI groups.

The results indicated that both in TDAPEI and PEI (25 kDa) groups, tumor cells were successfully transfected and VEGF shRNA was transcribed (Figures 6, 9). Then siRNA was produced by Dicer, which inhibited VEGF expression and played the important role of anti-angiogenesis (34). The growth rate of the tumor decreased due to the lack of oxygen and nutrition (34). The *in vivo* transfection efficiency of TDAPEI group was similar to that in PEI (25 kDa) group.

**Figure 10:**
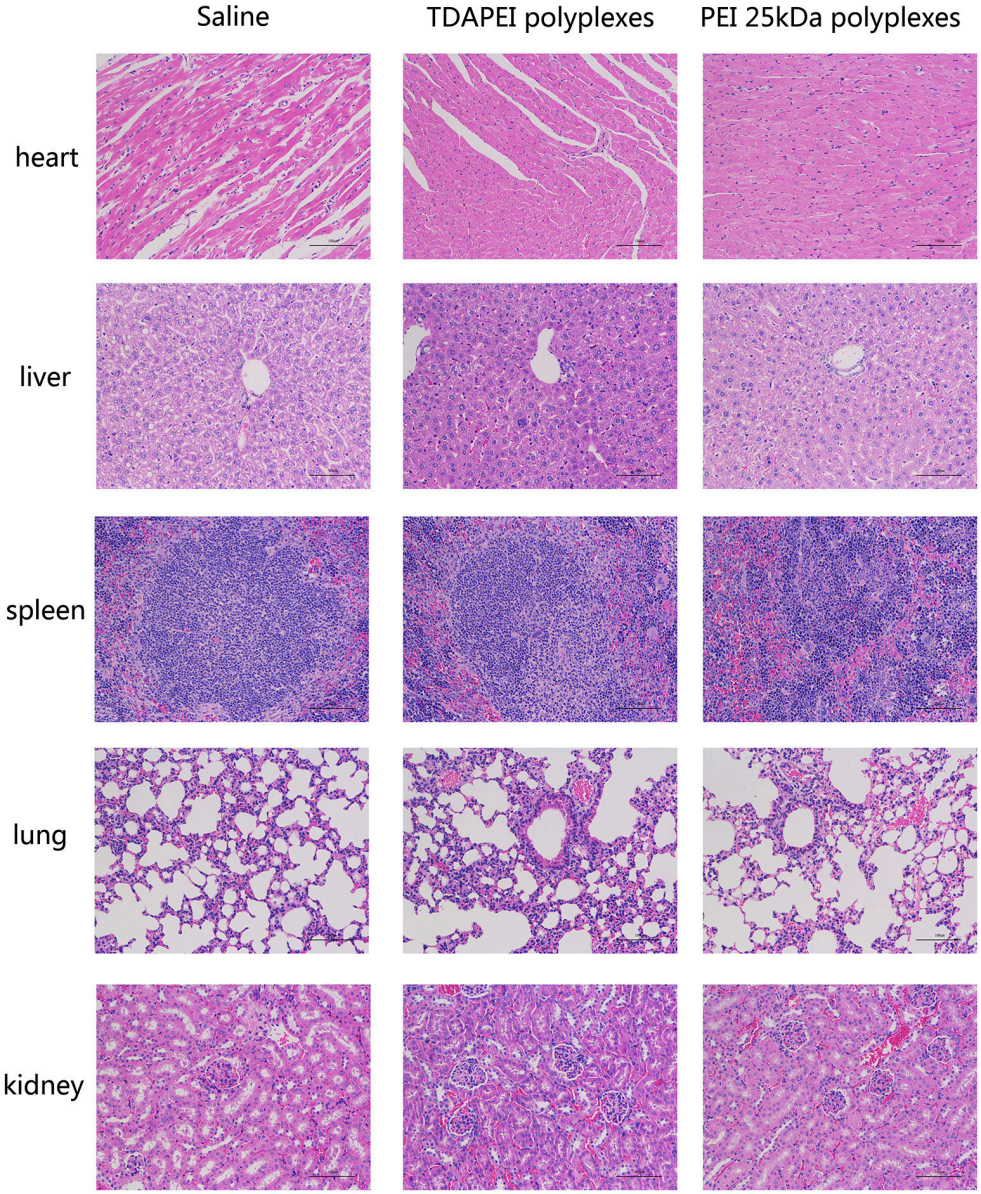
In vivo toxicity to main organs in saline, TDAPEI and PEI 25kDa groups measured by frozen section at Day 14. The organ sections were stained with hematoxylin and eosin (H&E).

Histological examination was adopted to demonstrate the toxicity of the polyplexes to organs *in vivo*. As is shown in Figure 10, almost no obvious lesion was observed in TDAPEI group compared to that in saline group, while in PEI (25 kDa) group, inflammation possibly occurred in the organs (34). A primary conclusion could be drawn that TDAPEI had negligible toxicity to the main organs.

## Conclusion

In general, the biodegradable polycation TDAPEI showed great potential as a non-viral gene vector for tumor treatment. TDAPEI had strong ability to condense pDNA into tight polyplexes to protect pDNA from nuclease. Particle sizes ranged from 110.5 to 183.4 nm and the zeta potential stabilized at about 27 mV, which was suitable for endocytosis. Compared to PEI (25 kDa), TDAPEI had a better performance both in cell and animal studies since the experimental results suggest its high transfection efficiency as well as significantly low toxicity. We believe it is owing to our design strategy that TDAPEI is biodegradable in low acidic medium and could be metabolized into low-molecular-weight PEI fragments, which was of low toxicity and could be eliminated by cells. All results in this article suggested that TDAPEI delivering plasmid DNA encoding anti-VEGF-shRNA may provide a promising method for tumor treatment.

## Author contributions

W-EY conceived the initial idea, the conceptualization, and the study design, and participated in the data extraction and analysis. YL, JC, and W-EY revised the manuscript. XL, XG, YC, XZ, ZF, YL, and JC and participated in the study design, searched databases, extracted and assessed studies and helped draft the manuscript. XL wrote the manuscript. All authors have read and approved the final manuscript.

### Conflict of interest statement

The authors declare that the research was conducted in the absence of any commercial or financial relationships that could be construed as a potential conflict of interest.
